# Cost-effectiveness of sacituzumab tirumotecan in previously treated metastatic triple-negative breast cancer in China

**DOI:** 10.1371/journal.pone.0343330

**Published:** 2026-03-06

**Authors:** Shuo Kang, Shuo Yang, Yize Jia, Shan Zhao

**Affiliations:** 1 Medical Insurance Office, The Second Hospital of Hebei Medical University, Shijiazhuang, PR China; 2 School of Pharmacy, Hebei Medical University, Shijiazhuang, PR China; 3 Department of Oncology, The Second Hospital of Hebei Medical University, Shijiazhuang, PR China; Hokkaido University: Hokkaido Daigaku, JAPAN

## Abstract

**Background:**

The OptiTROP-Breast01 trial demonstrated the efficacy of sacituzumab tirumotecan for patients with metastatic triple-negative breast cancer (TNBC). The current analysis evaluated the cost-effectiveness of sacituzumab tirumotecan compared with chemotherapy for patients with metastatic TNBC from the Chinese health-care system perspective.

**Methods:**

A partitioned survival model (PSM) was developed to simulate 3-week patients in 10-year time horizon to access the disease course and cost-effectiveness of sacituzumab tirumotecan compared with chemotherapy for metastatic TNBC patients, cost and utility values were gathered from the dataset and published studies, annual discount rate of 5% was used. Total cost, life-years (LYs), quality-adjusted life-years (QALYs), and incremental cost-effectiveness ratio (ICER) were the model outputs. Sensitivity analyses and subgroup analyses were conducted to estimate the robustness of the model outcomes.

**Results:**

In base-case analysis, compared with chemotherapy, sacituzumab tirumotecan could bring additional 0.41 LYs and 0.35 QALYs, with marginal costs of $55,927.31, resulting in the ICER of $162,799.04/QALY, which high than the willingness-to-pay (WTP) threshold of $40,326 per additional QALY gained. One-way sensitivity analyses revealed that the utility value was the main driver of the model outputs. Probabilistic sensitivity analyses showed the cost-effective probability of sacituzumab tirumotecan was 0% at the WTP threshold of $40,326/QALY. Subgroup analyses suggested that sacituzumab tirumotecan could not be considered cost-effective for all subgroup patients.

**Conclusion:**

Sacituzumab tirumotecan was unlikely to be the cost-effective option for patients with metastatic TNBC compared with chemotherapy from the Chinese health-care system perspective, reduced the price of sacituzumab tirumotecan could increase its cost-effective.

## 1. Introduction

Breast cancer is the most common type of malignant tumors among female with 2.3 million new diagnosed cases and 685,000 deaths in 2020 worldwide, which accounts nearly of 12% of new diagnosed cancers around the world [1]. The disability-adjusted life-years (DALYs) caused by breast cancer ranks first and accounts 17% in female malignant tumors reported by Global Burden of Disease (GBD) [2], and the 5-year survival rate of advanced breast cancer was only 20%. Triple-negative breast cancer (TNBC) is defined as negative for three relevant receptors among estrogen receptors (ERs), progesterone receptors (PRs), and human epidermal growth factors receptors 2 (HER2), TNBC accounts for 15%−20% of all breast cancers [3]. TNBC with the poor prognosis due to the poor response to endocrine treatment and targeted therapy, the rapid recurrence of the first-line therapy caused the median overall survival (OS) was 9–13 months for recurrent or metastatic TNBC [4]. With the limited treatments, chemotherapy remained as the standard therapy for patients with metastatic TNBC.

With the development of medical and health technology, the antibody drug conjugate (ADC) drug provides new treatment option for TNBC due to its powerful killing effect of tumor-targeting property. Sacituzumab tirumotecan is a novel ADC drug that targeting trophoblast cell surface antigen 2, which designed using a proprietary Kthiol linker to conjugate the topoisomerase I inhibitor KL610023 [5,6]. Recent randomized OptiTROP-Breast01 phase 3 clinical trial revealed the significant survival benefit of sacituzumab tirumotecan compared with chemotherapy for patients with metastatic TNBC (median progression-free survival [PFS]: 6.7 months versus 2.5 months, hazard ratio [HR] of 0.32, *P* < 0.00001) [7]. However, widespread use of the sacituzumab tirumotecan might bring the impact of the healthcare costs due to its high price, and we need pay more attention to “economic toxicity” of the high-value innovative drugs especially in resource-limited countries such as China, cost-effectiveness analysis was the important method to solve the above issue. The current analysis aimed to evaluate the cost-effectiveness of sacituzumab tirumotecan compared with chemotherapy for patients with metastatic TNBC from the perspective of Chinese health-care system.

## 2. Methods

### 2.1. Analytical overview and model structure

**Fig 1:**
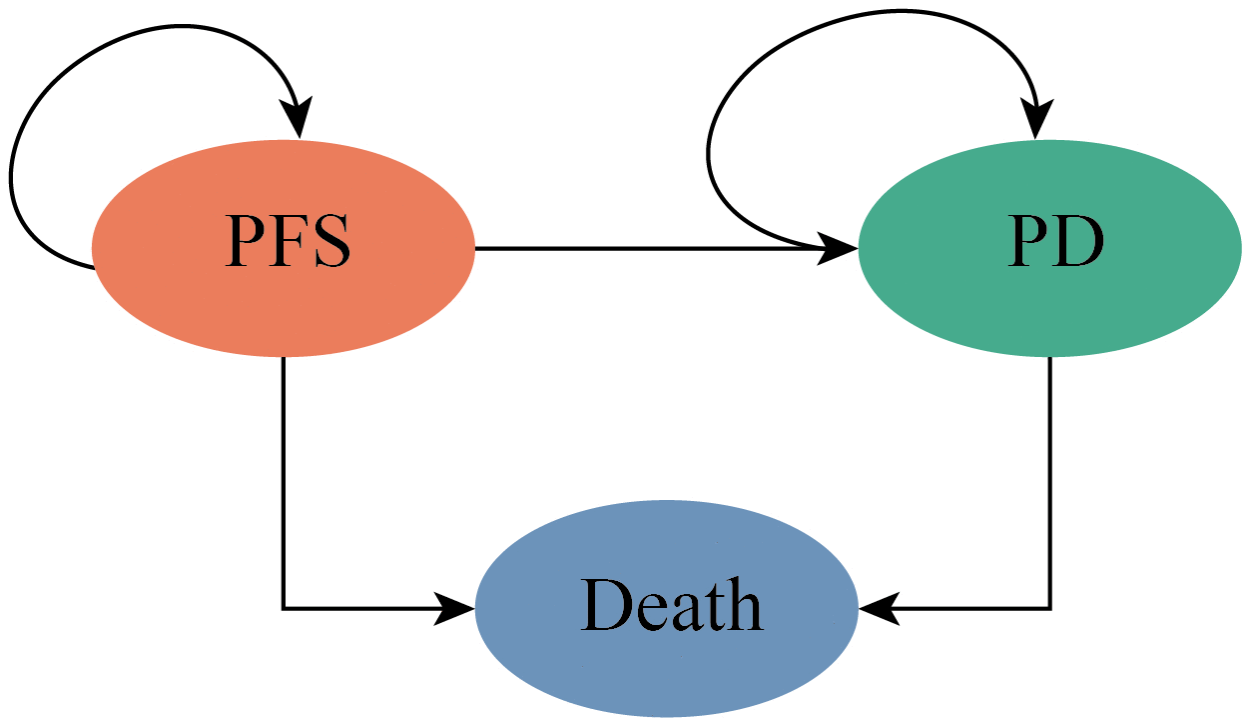
The structure of the partitioned survival model. PFS, progression-free survival; PD, progressed disease.

Partitioned survival model (PSM) was developed to estimate the health and economic value during 3-week patients’ transition in 10-year time horizon of the two competing strategies for patients with previously treated metastatic TNBC from the Chinese health-care system perspective. The PSM included three mutually exclusive health states: PFS, progressed disease (PD), and Death (Fig 1). Patients were at PFS state when entered the model, patients could not return to the previously health state, Death state was the absorbing state. The proportion of patients received subsequence treatment was obtained from the OptiTROP-Breast01 trial after the disease progressed.

The model outputs included total costs, life-years (LYs), quality-adjusted life-years (QALYs), incremental cost-effectiveness ratios (ICERs) were calculated by the formula: ICER=(C_2_-C_1_)/(E_2_-E_1_), and the incremental net-health benefits (INHBs) were also conducted based on the formula: INHB=(E_2_-E_1_)-(C_2_-C_1_)/λ, λ was the willingness-to-pay (WTP) threshold which was set as the three times of per capita gross domestic product (GDP) of China in 2024 ($40,326/QALY) in line with WHO recommendations [8–10]. All costs were converted into 2024 US dollars, costs and QALYs were discounted with the annual discount rate of 5% [11].

This study was based on the literature review and modeling techniques, so it did not require the approval by an institutional research ethics board.

### 2.2. Clinical data

Clinical outcomes used in the PSM were gathered from the OptiTROP-Breast01 trial. Long-term survival data beyond the observation period of the clinical trial were fitting by the standard methodology. First, we gathered the time and survival rate from the Kaplan-Meier (K-M) curves reported in the OptiTROP-Breast01 trial. Second, we reconstructed the individual time-to-event data by the algorithm developed by Guyot et al [12]. Then we used the time-to-event dataset to fit the parametric survival model among Exponential, Gamma, Generalized Gamma, Weibull, Log-normal, Log-logistic, and Gompertz [13]. Akaike information criterion (AIC) values and visual inspection were used to check the goodness of fitting, the AIC values of the fitting models were shown in Supplementary Table 1, the fitting curves of the K-M values were performed in S1 Fig. The survival parameters were shown in Table 1. Subgroup analyses were also conducted to evaluate the potential benefits in terms of cost-effectiveness of the competing treatments, the PFS and OS rates of sacituzumab tirumotecan were calculated by multiplying the PFS and OS rates of intention-to-treat patients in chemotherapy group and reported subgroup-specific HRs [14].

**Table 1:** Survival model parameters fitting to the PFS and OS data from the OptiTROP-Breast01 trial.

Treatment	PFS		OS	
	Model	Parameters	Model	Parameters
Sacituzumab tirumotecan	Log-normal	Meanlog = 1.7824; Sdlog = 0.9833;	Weibull	Shape = 1.67; Scale = 18.26
Chemotherapy	Generalized Gamma	Mu = 0.6801; Sigma = 0.6911; Q = −0.5723	Gamma	Shape = 2.2805; Rate = 0.1995

*PFS* progression-free survival, *OS* overall survival.

### 2.3. Cost and utility inputs

The current analysis was conducted from the perspective of Chinese health-care system, only direct medical costs were considered in the model. Costs inputs included anti-cancer drugs, best supportive care, routine follow-up, end-of-life care, and management of serious adverse events (SAEs, grade≥3). All costs values were obtained from the bid-winning price and published literatures, typical patient with height of 1.64m and weight of 65 kg, resulting the body surface area of 1.72m^2^ was used to calculate the dosage of the drugs [15].

Utility inputs which reflecting health state preference were obtained from the related studies. Health state utility values were on a scale of 0–1, the utility values of PFS, PD, and Death state were set to be 0.86, 0.6, and 0, respectively [16], in the current analysis, the disutility of SAEs were also included in the model, all costs and utility values were shown in Table 1.

### 2.4. Sensitivity analyses

One-way sensitivity and probabilistic sensitivity analyses were conducted to evaluate the robustness of the model results. In one-way sensitivity analyses, model parameters were changed one-by-one based on the lower and upper bounds to judge which was the most influence parameter of the model outputs. The range of each parameter was obtained from the reported or estimated 95% confidence intervals in the related studies. When the data was not available, assuming ±25% of the base-case line were used to be the lower and upper boundary (Table 2), the results of the one-way sensitivity analyses were shown by Tornado diagram. For probabilistic sensitivity analysis, Monte Carlo simulation of 1,000 iterations was conducted by repeatedly sampling the model parameters based on the statistical distribution, where Gamma distribution was used for cost inputs, Beta distribution was used for probabilities, proportions, rates, utility and disutility inputs, Log-normal distribution was used for subgroup- specific HR values [17], the results of probabilistic sensitivity analyses were performed by cost-effectiveness acceptability curves (CEACs).

**Table 2:** Model inputs: baseline value, ranges, and distribution.

Parameter	Baseline value	Ranges	Distribution	Reference
Cost inputs ($)				
Cost of sacituzumab tirumotecan per 200 mg	1319.5	989.63-1649.38	Gamma	Local charge
Cost of eribulin per 1 mg	98	73.5-122.5	Gamma	Local charge
Cost of capecitabine per 500 mg	3.16	2.37-3.95	Gamma	Local charge
Cost of gemcitabine per 1000 mg	30.59	22.94-38.24	Gamma	Local charge
Cost of vinorelbine per 10 mg	16.85	12.64-21.06	Gamma	Local charge
Cost of Nab-Paclitaxel per 100 mg	20.78	15.59-25.98	Gamma	Local charge
Cost of BSC per cycle	1449.53	1048.01-2071.46	Gamma	[23]
Cost of routine follow-up per cycle	161.4	121.09-201.86	Gamma	[24]
Cost of end-of-life care	1923.29	1442.47-2404.11	Gamma	[25]
Cost of neutrophil count decreased per event	104.95	78.71-131.19	Gamma	[26]
Cost of white blood cell count decreased per event	446.05	334.57-557.67	Gamma	[18]
Cost of platelet count decreased per event	150.08	112.59-187.56	Gamma	[18]
Cost of anemia per event	607.06	455.3-758.83	Gamma	[27]
Utility and disutility inputs				
PFS	0.86	0.645-1	Beta	[16]
PD	0.6	0.45-0.75	Beta	[16]
Disutility of neutrophil count decreased	−0.09	−0.11 to −0.0675	Beta	[28]
Disutility of white blood cell count decreased	−0.09	−0.1125 to −0.0675	Beta	[29]
Disutility of platelet count decreased	−0.19	−0.238 to −0.143	Beta	[30]
Disutility of anemia	−0.12	−0.15 to −0.09	Beta	[28]
Others				
Probability of neutrophil count decreased in sacituzumab tirumotecan group	0.346	0.264-0.428	Beta	[7]
Probability of white blood cell count decreased in sacituzumab tirumotecan group	0.277	0.200-0.354	Beta	[7]
Probability of platelet count decreased in sacituzumab tirumotecan group	0.131	0.073-0.189	Beta	[7]
Probability of anemia in sacituzumab tirumotecan group	0.292	0.214-0.370	Beta	[7]
Probability of neutrophil count decreased in chemotherapy group	0.47	0.385-0.555	Beta	[7]
Probability of white blood cell count decreased in chemotherapy group	0.364	0.282-0.446	Beta	[7]
Probability of platelet count decreased in chemotherapy group	0.038	0.006-0.070	Beta	[7]
Probability of anemia in chemotherapy group	0.061	0.020-0.102	Beta	[7]
proportion of patients received subsequence chemotherapy in sacituzumab tirumotecan group	0.508	0.422-0.594	Beta	[7]
Proportion of patients received subsequence chemotherapy in chemotherapy group	0.714	0.637-0.791	Beta	[7]
Discount rate	0.05	0-0.08	–	[11]

*BSC* best supportive care, *PFS* progression-free survival, *PD* progressed disease.

## 3. Results

### 3.1. Base-case analysis

In 10-year time horizon, sacituzumab tirumotecan could bring additional 0.41 LYs and 0.34 QALYs, with the marginal cost of $55,927.31, resulting in the ICER of $162,799.04/QALY (Table 3), which higher than the Chinese cost-effectiveness analysis WTP threshold of $40,326 per additional QALY gained, and it revealed that sacituzumab tirumotecan was unlikely to be the cost-effective treatment compared with chemotherapy for patients with previously treated metastatic TNBC from the Chinese health-care system perspective.

**Table 3:** The results of base-case analyses.

Treatment	Costs ($)	LYs	QALYs	ICER ($/QALY)
Chemotherapy	29,331.79	0.98	0.64	–
Sacituzumab tirumotecan	85259.09	1.39	0.99	162,799.04

*QALYs* quality-adjusted life-years, *ICER* incremental cost-effectiveness ratio.

### 3.2. Sensitivity analysis

**Fig 2:**
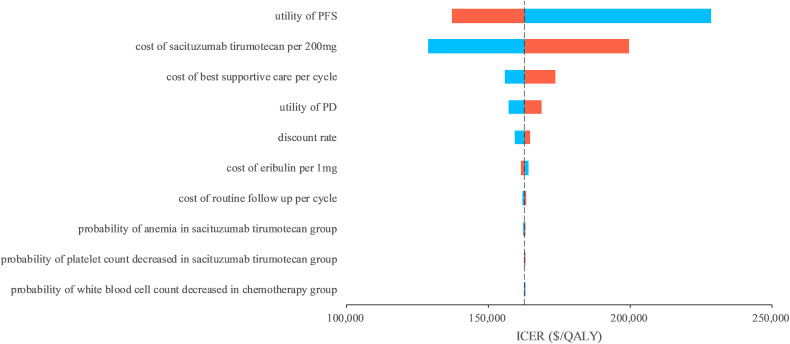
Tornado diagram of one-way sensitivity analyses of sacituzumab tirumotecan versus chemotherapy. PFS, progression-free survival; PD, progressed disease; ICER, incremental cost-effectiveness ratio; QALY, quality-adjusted life-year.

**Fig 3:**
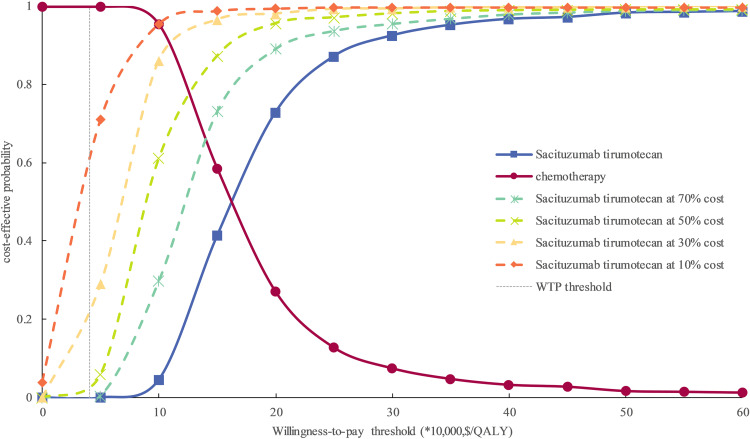
Cost-effectiveness acceptability curves of sacituzumab tirumotecan versus chemotherapy. WTP, willingness-to-pay; QALY, quality-adjusted life-year.

In one-way sensitivity analyses, the utility of PFS and the cost of sacituzumab tirumotecan was the most influence parameter of the model results (Fig 2), other parameters such as cost of best supportive care, utility of PD, and discount rate only have medium or minimal influences of the model outcomes, no matter how the parameters changed in one-way sensitivity analysis, the ICER remained higher that the WTP threshold of $40,326/QALY. In probabilistic sensitivity analyses, at the WTP threshold was $40,326/QALY, the probability of sacituzumab tirumotecan could be considered cost-effective was 0% compared with chemotherapy for patients with previously treated metastatic TNBC (Fig 3). To expanded the application scenarios of the model results, we conducted the exploration analysis when the price of sacituzumab tirumotecan reduced, we found that when the price of sacituzumab tirumotecan reduced 30%, 50%, 70%, and 90%, respectively, the cost-effective probability of sacituzumab tirumotecan was 0%, 2%, 17%, and 58%, respectively, and these findings were valuable for Chinese health-care decision makers for drug price negotiation.

### 3.3. Subgroup analysis

For subgroup patients, based on the subgroup-specific HRs of PFS and OS of sacituzumab tirumotecan compared with chemotherapy, the INHBs of sacituzumab tirumotecan compared with chemotherapy were always less than 0 for all subgroup patients, and the sacituzumab tirumotecan was unlikely to be the cost-effective treatment for all subgroup patients based on the subgroup probabilistic sensitivity analyses (S2 Table and S3 Table).

## 4. Discussion

With the unmet demand for treating metastatic TNBC, the satisfactory clinical results of sacituzumab tirumotecan compared with chemotherapy in OptiTROP-Breast01 trial interested both oncologist and health-care decision makers. However, the cost-effectiveness of sacituzumab tirumotecan for patients with metastatic TNBC was uncleared yet, especially when the price of sacituzumab tirumotecan was relatively expensive, the “economic toxicity” of the high-value innovative drug needs to pay more attention, which motivates the current analysis. The current analysis evaluated the cost-effectiveness of sacituzumab tirumotecan for patients with metastatic TNBC compared with chemotherapy. In base-case analysis, the ICER of sacituzumab tirumotecan versus chemotherapy was far higher than the WTP threshold of $40,326/QALY. One-way sensitivity analysis demonstrated that the utility value of PFS health state and the cost of sacituzumab tirumotecan have significant effect of the ICER. The probabilistic sensitivity analyses further confirmed the robustness of the base-case results, the cost-effective probability of sacituzumab tirumotecan at the WTP threshold of $40,326/QALY was 0%. These findings indicate that sacituzumab tirumotecan was unlikely to be the cost-effective option for patients with metastatic TNBC compared with chemotherapy in China.

At present, Chinese adopted the way of national medical insurance negotiation with pharmaceutical companies by cost-effectiveness evidence, and reducing drug price to improve the cost-effective was an important aspect, our study explored the scenario of the drug price reduction, and we found that when the price of sacituzumab tirumotecan reduced 90%, sacituzumab tirumotecan could be considered the cost-effective option for metastatic TNBC patients. A likely contributing factor to the results was Chinese government implemented volume-based procurement to reduce the price of non-innovative drugs such as chemotherapy. This policy guaranteed the market volume in exchange for drug price, it caused the price of chemotherapy similar to the non-competitive market conditions, resulting in lower costs of chemotherapy, consequently, the substantially larger price reduction was inevitable to improve the cost-effective of innovative treatment. Selected appropriate comparator therapy and strategically applied pharmacoeconomic are thus crucial challenges for Chinese government, effectively addressed these challenges is essential to optimize healthcare resource allocation efficiency while simultaneously maintained adequate incentives for pharmaceutical innovation.

To the best of our knowledge, the current analysis was the first study that evaluated the cost-effectiveness of sacituzumab tirumotecan compared with chemotherapy for patients with metastatic TNBC. Several recent studies evaluated the cost-effectiveness of sacituzumab govitecan, a similar ADC drug, compared with chemotherapy for metastatic TNBC patients in both China and US [18,19–22], these studies showed that sacituzumab govitecan was not the cost-effective option compared with chemotherapy in both China and US, the one-way sensitivity analyses demonstrated that the cost of sacituzumab govitecan and the utility of PFS state was the main driver of the model results, these findings were comparable and consistent with ours.

Several limitations must be illustrated when the model outcomes were used to make the health-care decision. First, long-term survival benefits beyond the follow-up time period in the OptiTROP-Breast01 trial were extrapolated by the mathematical fitting method, this method was an inevitable limitation of the anti-cancer drug economic evaluation that may cause bias between the model results and real-world data. Second, some cost value such as cost of best supportive care, cost of routine follow-up, and cost of end-of-life care were obtained from the previous studies rather than real-world data, however, one-way sensitivity analysis demonstrated the robustness of the model results when parameters were changed. Third, to simplify the model, only serious adverse events (SAEs, grade≥3) were involved in the model, which the results might be biased, however, the probability and cost of management of serious adverse events only have small influence of the model results revealed by the sensitivity analyses. Fourth, utility values which reflecting the health preference, and might be affected by region, race, and religious belief, in the current analysis, the utility values were gathered from the foreign study, although the utility of PFS state was the most influence factor of the model results, sensitivity analyses revealed that the model outcomes were robustness, and the change of ICER did not affect the cost-effective of sacituzumab tirumotecan. Finally, other potential competing treatments such as sacituzumab govitecan were not involved in the model due to the absence of the head-to-head trial. Despite these limitations, we adopted the standard methodological framework of the anti-cancer drug cost-effectiveness analysis in the current study. The model outcomes were robustness, and the bias of the analysis was within the controllable range, we were confident that the analysis accurately reflected the clinical conditions and the management of the metastatic TNBC in China, and our findings were valuable for Chinese health-care decision makers.

## 5. Conclusion

In conclusion, sacituzumab tirumotecan was unlikely to be the cost-effective option compared with chemotherapy for patient with metastatic TNBC from the Chinese health-care system perspective due to the unfavorable ICER, reduced the price of sacituzumab tirumotecan could increase its cost-effective.

## Supporting Information

S1 Table Summary of statistical goodness-of-fit of Kaplan-Meier curves in clinical trial. (DOCX)

S1 Fig The replicated Kaplan-Meier PFS and OS curves of the two competing regimens in OptiTROP-Breast01 trial. (DOCX)

S2 Table Subgroup analysis of incremental net health benefits (INHB) and probabilities of cost-effectiveness of sacituzumab tirumotecan versus chemotherapy by varying the hazard ratios (HRs) of progression-free survival (PFS). (DOCX)

S3 Table Subgroup analysis of incremental net health benefits (INHB) and probabilities of cost-effectiveness of sacituzumab tirumotecan versus chemotherapy by varying the hazard ratios (HRs) of overall survival (OS). (DOCX)
