# Supplementary material for: Cost-effectiveness of sacituzumab tirumotecan in previously treated metastatic triple-negative breast cancer in China
Source: PLoS One. 2026 Mar 6;21(3):e0343330. doi: 10.1371/journal.pone.0343330 (PMC12965532; doi:10.1371/journal.pone.0343330)
Supplement: S1 Table — (DOCX) [file pone.0343330.s001.docx]

**Supplementary table 1** Summary of statistical goodness-of-fit of Kaplan-Meier curves in clinical trial.

| Distribution | AIC | |
| --- | --- | --- |
|  | Sacituzumab tirumotecan | Chemotherapy |
| PFS | | |
| Exponential | 492.653 | 480.4538 |
| Gamma | 483.224 | 453.1436 |
| Generalized Gamma | 483.6051 | 428.4327 |
| Weibull | 484.2328 | 464.61 |
| Log-normal | 481.6348 | 432.3268 |
| Log-logistic | 484.5817 | 432.093 |
| Gompertz | 488.1341 | 481.2314 |
| OS | | |
| Exponential | 372.1182 | 521.3508 |
| Gamma | 363.1871 | 499.3916 |
| Generalized Gamma | 364.7279 | 501.3685 |
| Weibull | 362.7615 | 500.1466 |
| Log-normal | 366.4643 | 501.5834 |
| Log-logistic | 363.1026 | 499.4976 |
| Gompertz | 364.4444 | 506.586 |

AIC, Akaike information criterion; PFS, progression-free survival; OS, overall survival; *, adopted parametric survival function in the model.
