# Supplementary material for: Cost-effectiveness of sacituzumab tirumotecan in previously treated metastatic triple-negative breast cancer in China
Source: PLoS One. 2026 Mar 6;21(3):e0343330. doi: 10.1371/journal.pone.0343330 (PMC12965532; doi:10.1371/journal.pone.0343330)

**Supplementary figure 1** The replicated Kaplan-Meier PFS and OS curves of the two competing regimens in OptiTROP-Breast01 trial.


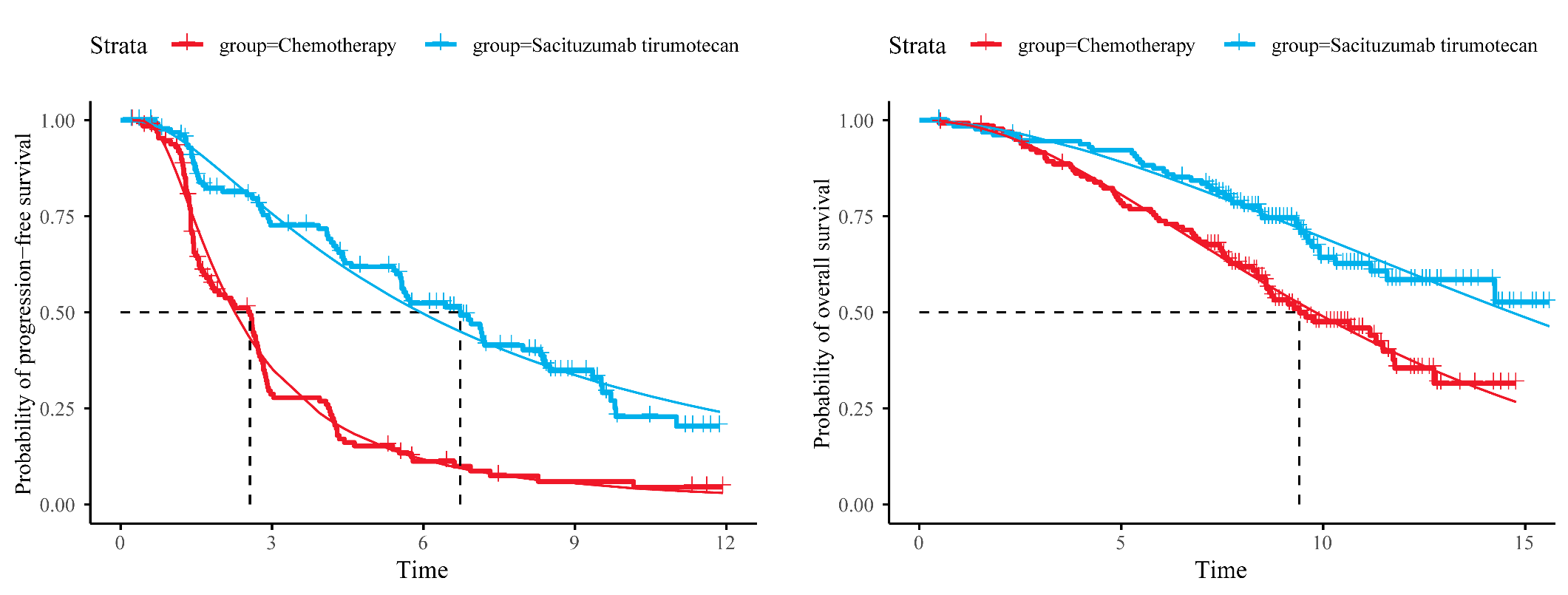

Supplement: S1 Fig — (DOCX) [file pone.0343330.s002.docx]
