# Supplementary material for: Cost-effectiveness of sacituzumab tirumotecan in previously treated metastatic triple-negative breast cancer in China
Source: PLoS One. 2026 Mar 6;21(3):e0343330. doi: 10.1371/journal.pone.0343330 (PMC12965532; doi:10.1371/journal.pone.0343330)
Supplement: S2 Table — (DOCX) [file pone.0343330.s003.docx]

**Supplementary table 2** Subgroup analysis of incremental net health benefits (INHB) and probabilities of cost-effectiveness of sacituzumab tirumotecan versus chemotherapy by varying the hazard ratios (HRs) of progression-free survival (PFS).

| **Subgroup** | **INHB (QALYs, median [Range])** | **Probability of cost-effectiveness** |
| --- | --- | --- |
| Age | | |
| <65 years | -1.31 (-1.51 to -1.06) | 0% |
| ECOG performance status | | |
| 0 | -1.19 (-1.54 to -0.73) | 0% |
| 1 | -1.22 (-1.46 to -0.92) | 0% |
| Prior line of therapy | | |
| 2 | -1.12 (-1.44 to -0.78) | 0% |
| 3 | -1.44 (-1.68 to -1.00) | 0% |
| >3 | -1.46 (-1.74 to -0.75) | 0% |
| Liver metastases | | |
| Yes | -1.19 (-1.54 to -0.78) | 0% |
| No | -1.29 (-1.54 to -0.98) | 0% |
| Initial diagnosis of TNBC | | |
| Yes | -1.15 (-1.41 to -0.85) | 0% |
| No | -1.36 (-1.64 to -0.92) | 0% |
| Prior treatment with PD-1 or PD-L1 inhibitors | | |
| Yes | -1.31 (-1.64 to -0.82) | 0% |
| No | -1.19 (-1.44 to -0.90) | 0% |
| Lymph node metastases | | |
| Yes | -1.19 (-1.46 to -0.87) | 0% |
| No | -1.24 (-1.54 to -0.84) | 0% |
| Visceral metastases | | |
| Yes | -1.36 (-1.56 to -1.10) | 0% |
| No | -1.19 (-1.71 to -0.46) | 0% |
| HER2 expression | | |
| Low expression | -1.22 (-1.54 to -0.81) | 0% |
| 0 | -1.29 (-1.54 to -0.96) | 0% |
| Course of disease (months) | | |
| <=24 | -1.17 (-1.51 to -0.75) | 0% |
| >24 | -1.26 (-1.51 to -0.96) | 0% |

INHB, incremental net health benefits; QALY, quality-adjusted life-year; ECOG, Eastern Cooperative Oncology Group; TNBC, triple-negative breast cancer; PD-1, programmed cell death protein 1; PD-L1, Programmed cell death ligand 1; HER2, Human Epidermal Growth Factor Receptor 2
