# Supplementary material for: Cost-effectiveness of sacituzumab tirumotecan in previously treated metastatic triple-negative breast cancer in China
Source: PLoS One. 2026 Mar 6;21(3):e0343330. doi: 10.1371/journal.pone.0343330 (PMC12965532; doi:10.1371/journal.pone.0343330)
Supplement: S3 Table — (DOCX) [file pone.0343330.s004.docx]

**Supplementary table 3** Subgroup analysis of incremental net health benefits (INHB) and probabilities of cost-effectiveness of sacituzumab tirumotecan versus chemotherapy by varying the hazard ratios (HRs) of overall survival (OS).

| **Subgroup** | **INHB (QALYs, median [Range])** | **Probability of cost-effectiveness** |
| --- | --- | --- |
| Age | | |
| <65 years | -1.08 (-1.11 to -1.03) | 0% |
| ECOG performance status | | |
| 0 | -1.06 (-1.12 to -0.93) | 1% |
| 1 | -1.07 (-1.11 to -1.02) | 0% |
| Prior line of therapy | | |
| 2 or 3 | -1.07 (-1.10 to -1.02) | 0% |
| >3 | -1.09 (-1.18 to -0.94) | 7% |
| Liver metastases | | |
| Yes | -1.05 (-1.10 to -0.94) | 0% |
| No | -1.08 (-1.12 to -1.03) | 0% |
| Initial diagnosis of TNBC | | |
| Yes | -1.07 (-1.11 to -1.01) | 0% |
| No | -1.07 (-1.12 to -0.96) | 1% |
| Prior treatment with PD-1 or PD-L1 inhibitors | | |
| Yes | -1.10 (-1.17 to -1.03) | 3% |
| No | -1.06 (-1.10 to -1.00) | 0% |
| Lymph node metastases | | |
| Yes | -1.07 (-1.11 to -1.02) | 0% |
| No | -1.08 (-1.13 to -0.97) | 1% |
| Visceral metastases | | |
| Yes | -1.07 (-1.10 to -1.02) | 0% |
| No | -1.12 (-1.27 to -0.91) | 22% |
| HER2 expression | | |
| Low expression | -1.09 (-1.13 to -1.02) | 0% |
| 0 | -1.05 (-1.10 to -0.97) | 0% |

INHB, incremental net health benefits; QALY, quality-adjusted life-year; ECOG, Eastern Cooperative Oncology Group; TNBC, triple-negative breast cancer; PD-1, programmed cell death protein 1; PD-L1, Programmed cell death ligand 1; HER2, Human Epidermal Growth Factor Receptor 2
